# Explorative Detection of Fractional Exhaled Nitric Oxide (FeNO) in Exhaled Breath of Patients With Breast Cancer

**DOI:** 10.1002/cam4.71279

**Published:** 2025-09-29

**Authors:** Francesco Segrado, Alessio Polymeropoulos, Michela Bianchi, Chiara Veronese, Roberto Agresti, Gianfranco Scaperrotta, Roberto Boffi, Rosalba Miceli, Rosaria Orlandi

**Affiliations:** ^1^ Nutrition Research and Metabolomics Unit, Department of Experimental Oncology Fondazione IRCCS Istituto Nazionale dei Tumori di Milano Milan Italy; ^2^ Biostatistics for Clinical Research, Department of Epidemiology and Data Science Fondazione IRCCS Istituto Nazionale dei Tumori di Milano Milan Italy; ^3^ Pulmonology Unit Fondazione IRCCS Istituto Nazionale dei Tumori di Milano Milan Italy; ^4^ Integrated Breast Surgery, Department of Surgical Oncology Fondazione IRCCS Istituto Nazionale dei Tumori di Milano Milan Italy; ^5^ Breast Imaging, Department of Diagnostic Imaging and Radiotherapy Fondazione IRCCS Istituto Nazionale dei Tumori di Milano Milan Italy

**Keywords:** breast neoplasms, breath tests, early detection of cancer, fractional exhaled nitric oxide testing, nitric oxide

## Abstract

**Background:**

Nitric oxide (NO), a gaseous messenger with pleiotropic functions, plays a role in cancer, including breast cancer (BC). Considering the high permeability and leakiness of NO across tissues and the increased levels of NO recently reported in exhaled breath and blood of patients with lung cancer, we explored exhaled NO levels in patients with BC in a future perspective of non‐invasive cancer detection.

**Patients and Methods:**

Fractional exhaled NO (FeNO) levels were detected in the breath of 192 women with BC and malignancy‐free controls employing a widely used point‐of‐care (POC)‐based system previously developed for asthma monitoring.

**Results:**

FeNO levels were lower in BC patients compared to controls, with the lowest levels in women with HER2‐expressing tumors. In univariate and multivariate analyses and after adjustment for age, smoking, and asthma, this difference was not significant. The effects of smoking were not statistically significant, whereas asthmatic subjects had significantly higher levels of FeNO (*p* = 0.006). Neither menopause nor BMI had a significant impact on FeNO levels.

**Conclusion:**

Our explorative work indicates that FeNO levels are heterogeneously detected in the breath of BC patients in the absence of confounding effects and are associated with the clinical characteristics of the disease. More sensitive detection of exhaled NO and larger cohorts enriched with ER negative BC are needed to further explore the potential of NO in non‐invasive detection of BC, either alone or in conjunction with other BC‐related volatile markers, and extending the NO measurement to blood or tissues.

**Abbreviations**
BC: breast cancer
BI‐RADS: breast imaging‐reporting and data system
BMI: body max index
ER: estrogen receptor
FeNO: fractional exhaled nitric oxide
HER2: human epidermal growth factor receptor 2
NOS: Nitric oxide synthases
POC: point‐of‐care
PR: progesterone receptor
TNBC: triple negative breast cancer
VOCs: volatile organic compounds

## 1 Introduction

Recent research in cancer biomarkers is focusing on the translational potential of volatile compounds in breath and bodily fluids as novel non‐invasive biomarkers for early cancer detection [1, 2]. Inorganic and organic volatile compounds are produced and released from the metabolic machinery of cancer cells and contain significant information on the biological process of cancer development and progression [3, 4].

Nitric oxide (NO) is a well‐known volatile biomarker applied to medical diagnosis. It is a highly reactive free radical and a multifunctional gaseous messenger molecule [5] that regulates vascular tone [6] and plays a key role as a cardiovascular signaling molecule [5, 7]. NO exerts pleiotropic functions in neoplastic transformation and progression, acting on cancer cells as well as cells and vessels of the tumor microenvironment. Alterations of biological activity and tissue levels of NO and nitric oxide synthases (NOSs), the enzymes responsible for the endogenous production of NO, have been reported in many tumors including breast and lung cancers [8, 9]. In the 1990s, exhaled NO was detected in human breath [10] and, accordingly, the fraction of exhaled nitric oxide (FeNO) became the first volatile biomarker developed for clinical diagnosis of respiratory diseases [11, 12]. More recently, NO levels were found to be higher in patients with lung cancer than in control subjects, suggesting that NO may contribute to the diagnosis of lung cancer and evaluation of therapeutic effects [13].

Breast cancer (BC) is the most commonly diagnosed cancer in women and is a leading cause of cancer‐related mortality worldwide [14]. Early and effective diagnosis by imaging techniques has always been a critical challenge for radiologists and oncologists. BC screening programs for women without signs of disease have been active since the 1970s and have led to the identification of cancer at an earlier stage than in symptomatic patients, ensuring a more treatable disease [15, 16]. Despite the efficacy of imaging modalities in detecting the disease, a clinical need in BC diagnosis remains the non‐invasive diagnosis of suspicious lesions that is currently addressed by invasive biopsy modalities. The efficacy of BC imaging is even suboptimal in detecting cancer in dense breasts, with a prevalence of overdiagnosis and false positives, especially among younger women, including high‐risk young women [17, 18].

For these reasons, there is a growing need to develop non‐invasive strategies that can support imaging techniques in the early detection of BC. Artificial Intelligence (AI)‐supported mammography screening resulted in a similar cancer detection rate compared with standard double reading [19], suggesting AI is a valuable tool for improving the quality and effectiveness of BC imaging. The complementation of digital imaging techniques with non‐invasive biological markers is today a unique opportunity to increase the specificity of BC detection in a common effort to reduce unnecessary invasive procedures and design a personalized BC diagnosis stratified by BC risk. This clinical need has fostered the identification of non‐invasive BC‐associated molecules, with a focus on metabolites featured in tissues and non‐invasive fluids such as exhaled breath [2].

Detection of volatile compounds offers an untapped potential for clinical diagnosis of cancer as the human volatome includes thousands of volatile molecules that are mostly unknown or not characterized [1]. Breath analysis is a novelty in medicine and has the potential to reshape clinical diagnosis due to its non‐invasiveness, painlessness, safety, ready acceptance by patients, and nearly unlimited access to samples [2, 20]. Application of breath analysis in clinical practice is currently achieved with the detection of a single volatile molecule, as in the case of exhaled nitric oxide for respiratory diseases [10, 11, 12] and *Helicobacter pylori* infection in gastroduodenal disease [21], but its application in cancer research is scaling up to global profiling of volatile metabolites from exhaled breath, fostering research/clinical studies aimed at identifying volatile compound signatures associated with many tumor types, including BC [20, 22, 23].

Detection and measurement of VOCs can be carried out by mass spectrometry (MS)–based techniques [2, 20] or using an electronic nose (e‐nose) that can be developed into point‐of‐care (POC) instrumentation for a rapid and straightforward non‐invasive diagnosis [20, 24]. POC testing provides indeed instant or rapid results at or near the patient's location, decreasing the time to diagnosis and facilitating clinical decision making in common disease processes. This approach to diagnostic medicine reduces requirements for specialist clinical staff and improves cost‐effectiveness. In oncology, POC technology would provide a simple solution to the measurement of volatile compounds and is a promising alternative to complex and expensive MS‐based technologies, but is currently waiting for robust volatile biomarkers validated in large clinical studies [22].

E‐nose devices are classified primarily by their sensing technology, which uses different types of chemical sensors to detect volatile compounds with metal oxide semiconductors (MOS) [25, 26]. POC technology has made significant progress in breath analysis, especially through advancements in signal processing and hardware engineering that have led to the development of portable POC devices with improved sensitivity and specificity employed to detect non‐cancer diseases as well as cancer [27, 28].

The technological complexity of breath analysis and the amount of breathomics data from a large number of healthy and diseased subjects has recently promoted the use of machine learning algorithms and other AI‐based methodologies to enhance classification accuracy and efficiency in detecting cancer [29, 30, 31, 32]. The integration of breathomics, novel analytical techniques, and AI represents a promising non‐invasive approach for early cancer detection.

Considering that NO has a role in cancer progression [8, 9] and is a gaseous, lipophilic, and extremely diffusible molecule detectable in tissues and exhaled breath, approaches to BC cancer diagnosis by the non‐invasive detection of NO are very promising, especially through imaging techniques and breath analysis and using NO alone or, in a future perspective, in conjunction with other BC‐related volatile markers. Novel imaging agents are under development for non‐invasive detection of NO and NOSs by optical approaches, electron paramagnetic or magnetic resonance, and positron emission tomography (PET) [33]. Recently, an imaging approach for monitoring the immune responses after immunotherapy has been proposed that detects NO release by tumor‐associated macrophages using a NO‐induced fluorescent probe [34]. In breath analysis, besides the current use of FeNO in the clinical diagnosis of respiratory diseases [11, 12], novel approaches using NO as a potential cancer biomarker are addressed to non‐invasive detection of lung cancer [13]. A commonly used portable detection system based on an electrochemical sensor that measures FeNO [35] is now commercially available for the diagnosis and management of asthma [12], whereas novel sensors for NO detection are in development [36].

Herein, we evaluated FeNO levels in a series of 192 patients with BC and malignancy‐free controls to assess a non–invasive approach that would explore a clinical role of NO in the detection of BC. The present research is specifically addressed to clinical practice and applies the commercially available POC device commonly used in medical diagnostics to detect FeNO in the perspective of a straight translational application. This approach supports the growing field of volatile compounds in cancer diagnosis, addressing the BC diagnostic need for non‐invasive volatile biomarkers able to complement digital imaging techniques with the aim of improving diagnostic accuracy and reducing invasive procedures.

## 2 Patients and Methods

### 2.1 Study Design and Participant Recruitment

The present study was designed as a prospective study at the Fondazione IRCCS Istituto Nazionale dei Tumori (INT) and was approved by the Ethics Committee of the same institution (INT 69/18). The study design applied a consecutive recruitment strategy to address the complexity and heterogeneity of BC and achieve a comprehensive understanding of outcomes and limitations of the FeNO detection methodology.

The sample size of the cohort was based on practical considerations and reflected the explorative nature of the present study.

Women were consecutively recruited and sampled from June 2022 to April 2023 among BC patients who underwent breast surgery on the day of pre‐operative testing. Malignancy‐free controls were screened at the Breast Imaging Unit. The following inclusion criteria were applied: age ≥ 18 years and signed informed consent; primary BC at an early stage for patients and breast imaging‐reporting and data system (BI‐RADS) = 1 (negative) or 2 (benign with 0% probability of malignancy) for control subjects [37]. Exclusion criteria included the following: concomitant acute infection or other serious concomitant medical disorders, oral disorders, concomitant treatment with immunosuppressive or immunomodulating drugs, and a history of cancer in the past 5 years. Breath collection and analysis of patients with BC were assessed at the diagnosis and before surgery, in the absence of concurrent pharmacological or radiotherapy‐based anti‐tumor treatments.

Study participants were supplied with a leaflet explaining the aims of the study and providing technical information. They were asked not to smoke, eat, drink (except for water), brush their teeth, or use lipstick for at least 2 h before breath analysis, and measurements were performed in accordance with the clinical indications for FeNO testing to diagnose asthma, chronic obstructive pulmonary disease (COPD), and lung diseases [12, 38, 39, 40]. A questionnaire collecting information on personal data, medical history, and lifestyle was completed prior to sampling to control individual factors that may influence exhaled NO measurements.

Clinical–pathological data of the patient group were retrieved from the institutional clinical database, including ER, PR, HER2, and Ki‐67 status. Sub‐typing of BC samples was assessed according to immunohistochemical determination of hormonal receptors, HER2, and Ki‐67 as follows: Luminal A (ER+ and/or PR+, HER2−, Ki‐67 < 20%); Luminal B (ER+ and/or PR+, HER2−, Ki‐67 ≥ 20%); HER2+ (HER2+, any Ki‐67). Patients carrying TNBC (ER−, PR−, HER2−, any Ki‐67) were not included in the series of BC patients, mainly because they were under‐represented in the consecutive recruitment and not compliant with the inclusion criteria as they are generally treated with neoadjuvant therapy before surgery.

### 2.2 NO Measurements in Exhaled Breath

The fractional NO concentration in exhaled breath (FeNO) was measured by FeNObreath (Bedfont Scientific Ltd) according to the manufacturer's directions (https://fenobreath.bedfont.com/wp‐content/themes/fenobreath/assets/documents/manuals/FeNObreath‐Manual‐UK.pdf) and the international guidelines for online NO measurement [12, 39, 41].

The electrochemical sensor underwent calibration procedures on a regular basis to ensure FeNO measurement accuracy throughout the study. Since the main critical factors in ensuring reproducible and standardized measurements of FeNO are the exclusion of nasal NO and the standardization of exhalation flow [38], no nose clip was used and an exhalation flow of 50 mL/s was maintained for each participant. According to the manufacturer's instructions, participants were asked to inhale deeply, hold their breath for 15 s, and then blow steadily into a disposable mouthpiece supplied with the device. The exhalation was guided by an on‐screen flow meter, so participants could provide a controlled exhalation flow. It takes some seconds for the flow to stabilize, so the dead space air does not affect the FeNO measurement as it is completely expelled before the measurement begins. In the case that the flow deviates from the 50 mL/s ±10% set point, the measure was automatically interrupted by the device and the participant was asked to repeat the test. FeNObreath allowed a controlled flow at 10 cm H_2_O so that the resultant mouthpiece pressure ensures velum closure during exhalation and excludes contamination of the exhalate sample with nasal NO [39]. FeNO was measured in duplicate. An interval of more than 1 min was used between repeated measurements, in order to get participants ready for the next test. This pause was considered sufficient to reduce potential carry‐over effects. FeNO concentrations were expressed in parts per billion (ppb). When duplicate measurements showed discrepancies over the repeatability threshold declared by the user manual (±5 ppb of measured value ≤ 50 ppb or ±10% of measured value > 50 ppb), the test was repeated.

### 2.3 Safety Procedures

FeNO sampling was carried out by adopting a risk‐reduction approach in order to avoid the spread of SARS‐CoV‐2 or other infective agents [42]. A sampling room only accessible to authorized personnel and equipped with a HEPA filter air purification system was used for sampling. Before interacting with subjects, operators wore nitrile gloves, a disposable waterproof gown, and FFP2 respiratory masks. All participants were free of acute infection of respiratory tract and were vaccinated against SARS‐CoV‐2.

### 2.4 Smoking Definitions and Parameters

Never smokers or subjects who stopped smoking for at least 1 year were considered non‐smokers, whereas all the others, independently of smoking intensity, were considered active smokers. E‐cig or heated tobacco products were not used by smokers or former smokers. Self‐reported smoking habits were used to assess tobacco smoke exposure. Smokers were asked about the average daily number of cigarettes smoked and the total number of smoking years, which were used to calculate the number of pack‐years as follows: number of cigarettes smoked per day/20 × number of smoking years.

### 2.5 Statistical Analysis

Data were evaluated using standard descriptive statistics, and hypothesis testing was applied to assess the strength of association of FeNO levels in BC patients and controls. For continuous variables, summary statistics were calculated using the mean, median, range [min–max], and SD, whereas for categorical variables, frequency count (*N*) or percentage (%) was used as appropriate. Correlation between continuous variables was based on Pearson's or Spearman's correlation coefficient [43]. Group associations with continuous variables were assessed based on parametric and non‐parametric statistical tests. In particular, parametric tests were based on analysis of variance (ANOVA or t‐test) using mean comparison, whereas in the case of non‐parametric tests, Wilcoxon was used for median comparison [44, 45]. In addition, to ensure the validity of parametric tests, Levene's test for homoscedasticity of variance [46], normality tests based on Shapiro–Wilk [47], and quantile plots (qqplots) are reported on each stratum of categorical variables [48]. Normality tests and quantile plots were done after removing any influential point based on Grubbs' test [49]. Continuous variables were also transformed to log scale to correct for skewness and meet the criteria for statistical tests. Finally, a linear regression model was implemented to adjust for potential confounding effects of other clinical variables [50]. Analysis of residuals was also provided to satisfy the requirements of linear regression.

Statistical analyses were carried out using R software, version 4.3.2 (www.r‐project.org/). Differences were considered significant at *p*‐values < 0.05.

## 3 Results

### 3.1 Characteristics of Study Participants

**FIGURE 1:**
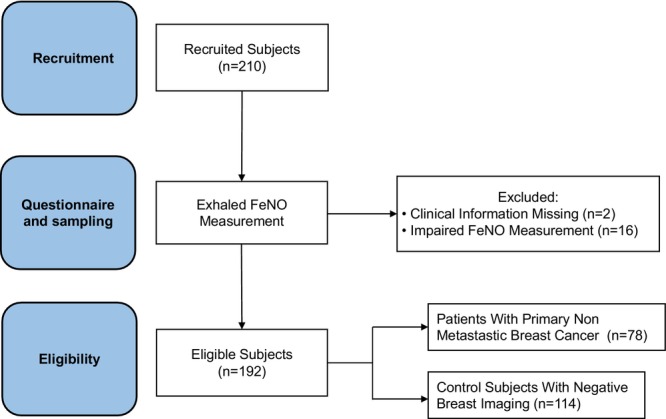
Study flow chart outlining the number of participants recruited, sampling, data selection, and groups compared in statistical analysis. The questionnaire included date of birth, weight, and height, menopause state, history of asthma, smoking status, and type of nutrition. Outcome measures of the study were evaluation of possible confounder factors affecting FeNO measurements and comparison of FeNO levels in breast cancer and control groups.

Women with or without BC were consecutively recruited and subjected to FeNO measurements (Figure 1). FeNO values and demographic, medical, and lifestyle characteristics of the eligible women, 78 with an early‐stage BC and 114 control subjects with negative BC imaging, are detailed in Table S1 and summarized in Table 1. Sixteen women did not complete the breath test (7.6%) since they were unable to maintain a stable exhalation rate.

**TABLE 1:** Characteristics and FeNO levels of study participants.

*N* = 192	Breast cancer	Controls
	*N* = 78	*N* = 114
Age (years)		
Mean	58.9	56.3
Median	57.5	56
Range (Min–Max)	24–86	22–85
Menopause		
No	29 (37%)	32 (28%)
Yes	49 (63%)	82 (72%)
BMI (kg/m^2^)		
Mean	23.7	23.1
Median	23.2	22.8
Range (Min–Max)	16.1–34.9	17.5–33.3
Smoking		
No	46 (59%)	64 (56%)
Yes	7 (9%)	14 (12%)
Smokers—Cigarette use (mean)		
Cigarettes/Day	7.2	5.4
Smoking years	33.7	31.8
Pack/years	10.5	8.8
Former smokers	25 (32%)	36 (32%)
Former smokers—Cigarette use (mean)		
Cigarettes/Day	15.1	10.1
Smoking years	19.6	18.5
Pack/years	15.6	10.8
Asthma		
No	78 (100%)	107 (94%)
Yes	0 (0%)	7 (6%)
Diabetes		
No	72 (92%)	109 (96%)
Yes	6 (8%)	5 (4%)
Hypertension		
No	57 (73%)	86 (75%)
Yes	21 (27%)	28 (25%)
FeNO_Avg (ppb)		
Mean	19.2	20.5
Median	15	15.5
Range (Min–Max)	3.5–217	6–100

A questionnaire administered to each participant before sampling was used to collect personal and anthropometric data, lifestyle factors (including smoking and dietary habits), and medical history, which was implemented by the institutional clinical database and was focused on chronic diseases such as asthma, hypertension, metabolic disorders such as diabetes, and the chronic use of medications.

The age of study participants ranged from 22 to 86 years with a mean of 58.9 for patients and 56.3 for controls, and thus most of the women were in post‐menopause (68%). All women generally followed a Mediterranean diet, most with a reduced consumption of red meat. BMI ranged from 16.1 to 34.9 kg/m^2^ with a mean of 23.7 for patients and 23.1 for controls, respectively. The series is characterized by a low proportion of smokers (11%) compared to a smoking prevalence of 18%–23% among women in Italy [51]. There were 21 smoker women and 61 former smokers who were considered non‐smokers. Mean smoking history was 9.3 and 12.7 pack/years for smokers and ex‐smokers, respectively; ex‐smokers had quit smoking from 1 to 50 years before breath sampling. There were 7 women with asthma in the control group.

Less than 8% of women had diabetes, whereas hypertension and use of antihypertensive drugs were registered in the 26% of women, and depended on age [52]; both diabetes and hypertension were balanced in the BC and control groups.

The prospective consecutive series of BC included 10% in situ carcinoma and, among invasive carcinomas, 43% Luminal A, 40% Luminal B, and 7% HER2 (Table S1). As expected in a consecutive recruitment of BC patients, the most represented subtypes are the luminal ones, whereas the estrogen‐negative subtypes like HER2‐positive and TNBC are significantly infrequent, with TN definitely representing the least common BC subtype. In our cohort, TNBC cases were not included due to their low frequency and the modality of patient recruitment. Women with BC were selected among those undergoing breast surgery on the day of pre‐operative testing, but women with TNBC were generally treated with neoadjuvant therapy and did not meet at surgery the inclusion criteria of the absence of treatment required in the present study.

### 3.2 Analysis of Factors Potentially Influencing FeNO Measurements

The FeNO measurements detected in our series of women were below the value of 25 ppb (79%), in the range of normal physiological values in adults, according to FeNO levels of 25–50 ppb detected in large population studies [12, 39, 41]. FeNO values > 50 ppb were 4% of samples, as expected [38], and were not related to the features of study participants listed in Table S1. Measurements were performed in duplicates, and FeNO mean values of replicates with their standard errors are shown in the Figure S1. No significant difference was found between the first and second replicate (Figure S2).

A significant influence of sex, age, BMI, and smoking on exhaled breath content has been recently reported in a large cohort of volunteers subjected to breath analysis [53]. In particular, sex, age, ethnicity, BMI, and genetic factors were identified as factors potentially affecting FeNO levels [12, 54]. Sex and smoking have been indicated as confounders of FeNO detection in patients with COPD [55].

**FIGURE 2:**
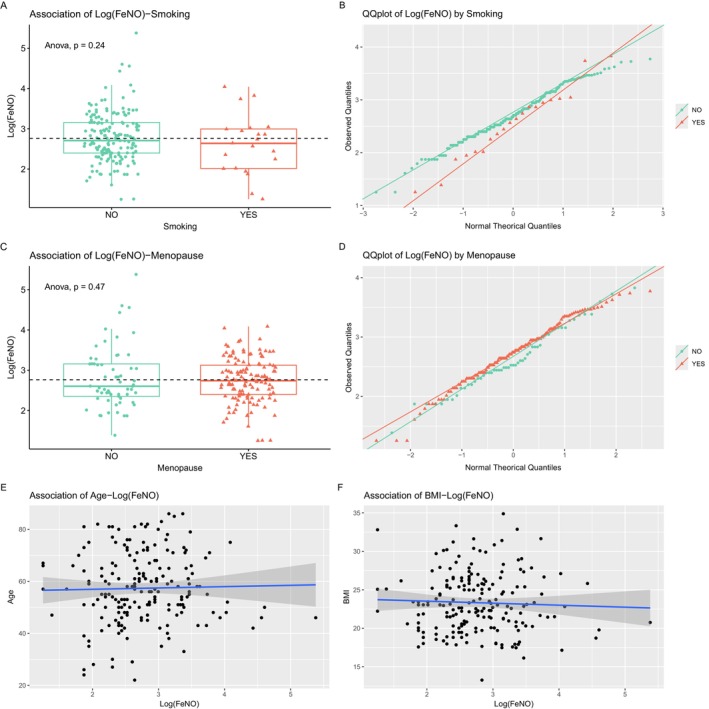
Analysis of potential confounders of FeNO measurements. Log(FeNO) levels in the exhaled breath of non‐smoker versus smoker women (A) and in pre‐ and post‐menopause women (C). *p* Values of ANOVA tests are reported in the graphs, and the relative QQ plots are shown (B, D). The association of Log(FeNO) levels with age and BMI is reported in (E) and (F), respectively.

In our series of women, no significant difference was found in mean and median FeNO levels in smokers versus non‐smokers (mean: *p* = 0.24, median: *p* = 0.32, Figure 2A). Mean and median FeNO values in log scale were 2.61 ± 0.73 and 2.64 ± 0.73 in smokers, respectively, and 2.78 ± 0.61 and 2.71 ± 0.61 in non‐smokers, respectively. Homogeneity of variance in smokers versus non‐smokers was satisfied (*p* = 0.27), and the normality assumptions together with QQ plots were valid for smokers (*p* = 0.75) and non‐smokers (*p* = 0.12) after checking outliers (Figure 2B). Table S2 lists the outliers determined by Grubbs test.

Menopause did not seem to influence the measurement of exhaled NO as shown in Figure 2C (mean: *p* = 0.47, median: *p* = 0.74). No significant difference was found in mean and median FeNO levels among post‐menopause versus pre‐menopause women. Mean and median FeNO levels on a log scale were 2.74 ± 0.55 and 2.74 ± 0.55 in post‐menopausal women, respectively, and 2.81 ± 0.74 and 2.60 ± 0.74 in pre‐menopause women, respectively. The homogeneity of variance in post‐menopause versus pre‐menopause women was not met at the borderline of significance, suggesting unequal variance among the two groups (*p* = 0.045). Normality assumptions were met based on QQ plots both for post‐menopause (*p* = 0.09) and pre‐menopause women (*p* = 0.48) after checking for outliers (Figure 2D, Table S2).

The association of FeNO levels with age was not statistically significant (rho_pearson = 0.02, *p* = 0.74 and rho_spearman = 0.04, *p* = 0.50, Figure 2E). Similarly, BMI did not seem to influence FeNO measurements (rho_pearson = −0.04, *p* = 0.56 and rho_spearman = −0.01, *p* = 0.85, Figure 2F).

### 3.3 FeNO Measurements in Patients With Breast Cancer and Malignancy‐Free Controls

**FIGURE 3:**
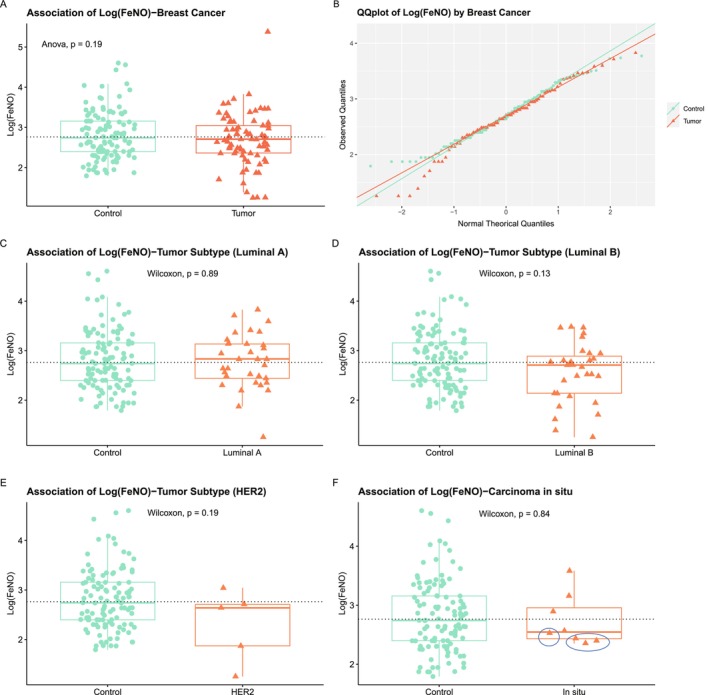
Analysis of FeNO levels in the exhaled breath of 192 women with and without breast cancer. Log(FeNO) levels in patients with breast cancer and controls (A). *p*‐Values of the ANOVA test are reported in the graph, and the relative QQ plot is shown (B). Log(FeNO) levels of breast cancer subtypes (C, D, and E) and in situ carcinomas (F) are compared with Log(FeNO) levels in controls. *p*‐Values of the Wilcoxon tests are reported in the graphs. In situ carcinomas that are HER2 positive are indicated by circles.

FeNO levels were compared in patients with BC and controls (Table 1 and Figure 3A). Statistical inference was conducted to assess the difference of breath levels of FeNO in patients with BC versus controls. The analysis was adjusted for the effect of smoking and age; asthma was not considered as only 7 control subjects were affected. No significant difference was found in mean and median FeNO levels (mean: *p* = 0.19, median: *p* = 0.36, Figure 3A). Mean and median of FeNO levels (log scale) were 2.69 ± 0.65 and 2.71 ± 0.65 in patients with BC, respectively, and 2.81 ± 0.60 and 2.74 ± 0.60, respectively, in controls. Equal variance test in BC cases versus controls was satisfied (*p* = 0.84), and the normality assumptions alongside QQ plots held for cases and controls (*p* = 0.13, *p* = 0.06, Figure 3B) after checking for influential points (Table S2). The FeNO levels in Luminal A, Luminal B, and HER2 BC subtypes were compared to FeNO levels in controls (Figure 3C–E, respectively). Luminal tumors showed no significant differences (Figure 3C,D). The median levels in exhaled NO were lower in women with HER2‐positive tumors (2.64 ± 0.72, Figure 3E) and in situ BC (2.55 ± 0.43, Figure 3F), although the differences were not significant. In in situ cases, the lowest FeNO levels were associated with the expression of the HER2 receptor within the carcinoma (Figure 3F).

**FIGURE 4:**
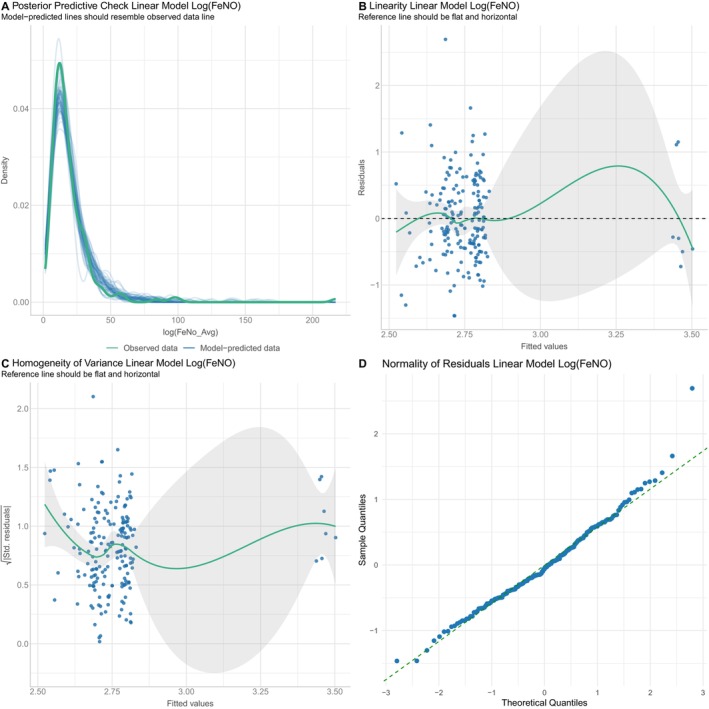
Assumptions of multivariate linear model investigating the association of Log(FeNO) in patients with breast cancer versus controls adjusted for age, smoking, and asthma. The assumptions of the linear regression model checked the model's adaptability by posterior predictive check test (A), linearity assumption (B), homogeneity of variance of the linear model (C), and the normality of residuals (D).

The difference in FeNO levels in patients with BC and controls was further investigated by adjusting for the effect of age, smoking, and asthma into a linear regression model with FeNO values as the outcome measure (Table 2). The results showed that on average Log(FeNO) levels of BC patients did not differ from controls even after adjustment for the three confounders (*p* = 0.338). The effect of smoking (*p* = 0.306) and age (*p* = 0.661) was not significant, whereas asthmatic subjects had significantly higher FeNO levels (*p* = 0.006). The assumptions of linear regression residuals were all satisfied (Figure 4A–D).

**TABLE 2:** Multivariate linear model investigating the association of Log(FeNO) in patients with breast cancer versus controls adjusted for age, smoking habit, and asthma.

Dependent variable Log(FeNO)			
Clinical variable	Estimates	95% CI (L, U)	*p*
Intercept	2.71	(2.32, 3.10)	< 0.001
BC versus controls	−0.09	(−0.27, 0.09)	0.338
Smoking habit (yes vs. no)	−0.15	(−0.43, 0.14)	0.306
Age	0.001	(−0.01, 0.01)	0.661
Asthma (yes vs. no)	0.67	(0.20, 1.15)	0.006

*Note:* *R* ^2^/*R* ^2^ adjusted: 0.058/0.037.

## 4 Discussion

Assuming that NO plays a role in BC biology and has been reported to contribute to BC progression and aggressiveness, especially in the more aggressive forms [8, 9], we explored a non‐invasive approach to detect NO levels in the breath of patients with BC. NO is a highly diffusible gas that can be detected in biological fluids and exhaled breath, and a fully developed POC technology for FeNO detection is currently available [11, 12]. Thus, the NO breath test has potential as a non‐invasive tool for diagnosis and monitoring in oncology, as highlighted by the recently reported increase of NO in the blood and breath of patients with lung cancer [13]. The accessibility of POC‐based technology, already developed and approved for the monitoring of respiratory diseases, represents a significant advantage for translational studies, providing reliable and simple measurements, as well as reduced time for testing. This approach meets with the clinical need of reshaping BC diagnosis towards a completely non‐invasive personalized diagnosis, tailored on BC risk of women and able to reduce unnecessary invasive procedures, with a great advantage especially for young and high‐risk women. The integration of digital BC imaging with noninvasive biomarkers represents an opportunity to improve the specificity and effectiveness of BC detection by imaging.

In the present explorative study aimed at evaluating the feasibility of FeNO detection in BC in a clinical context, we analyzed FeNO levels in the breath of 192 patients with BC and malignancy‐free women using a portable FeNO detector and clinical protocols for breath analysis, including safety measures for the pandemic. In our series of women, exhaled NO levels in patients with BC were lower compared to those in controls. This difference was not significant by univariate and multivariate analysis after adjustment for potential confounders such as age and smoking status. QQ plots and normality tests applied to FeNO data suggested that the results were consistent despite the limited sample size.

FeNO is a marker of type 2 inflammation in asthma, and increased FeNO levels are pathognomic of asthma exacerbation [12], although a decrease in FeNO has been reported in several diseased and non‐diseased conditions. FeNO levels are decreased in primary ciliary dyskinesia [56] and in cystic fibrosis [57], possibly associated with impairment of NOSs, and in patients with mitochondrial disorders [58]. In addition, decreased values of FeNO have been observed in smokers and ex‐smokers, with the lowest levels in smokers. A significant association of low FeNO with blood eosinophil or neutrophil levels has been found in ex‐smokers, but not in smokers [55]. In lung cancer, both an increase and a decrease of FeNO have been observed, depending on the tumor type. Non‐small cell lung cancer (NSCLC) is often associated with a higher inflammatory response and shows increased levels of exhaled NO. In contrast, small‐cell carcinoma (SCLC), a neuroendocrine tumor with low immune infiltration, is not associated with an increase in exhaled NO [13].

In our BC study, exhaled NO levels were specified in pre‐invasive “in situ” BC and in the intrinsic subtypes of invasive BC, taking into account the significant heterogeneity of the disease. The lowest levels of exhaled NO were detected in invasive and in situ HER2‐expressing tumors, but the small sample size requires independent confirmation in large studies.

Therefore, NO levels in the exhaled breath of BC patients seem to be associated with the clinical characteristics of BC, since the FeNO level decreases especially in HER2‐positive tumors. Interestingly, an experimental observation archived at the tissue level matches with our breath results, linking NO levels and HER2 expression. A reduced basal NO level has been reported to induce precancerous lesions that overexpress TGFβ and HER2 in mammary tissues. Consistently, increasing NO levels through the use of a NOS cofactor in precancerous and cancerous breast cells downmodulates TGFβ and HER2 and suppresses the proliferative phenotype [59]. However, further investigations are needed to understand the unknown mechanisms able to transfer the local metabolic interactions at a systemic level and the disease‐related changes due to the interaction between NO and the surrounding molecules in tissues that potentially impact breath composition.

Low FeNO levels in BC will require validation and future studies leading to the concomitant assessment of NO and its activities in tissues, blood, and breath within a large series of controls and BC patients, with a special focus on the association of NO with the immune microenvironment and systemic inflammatory markers of each BC subtype. This will provide new insights that will be useful to decipher the biological/clinical role of NO in cancer and establish the relevance and clinical utility of FeNO in BC detection.

Concerning the refinement of the FeNO test in BC, evidence from our data suggests that age, menopause, BMI, and smoking did not significantly impact FeNO levels, at least in our series of women. Nevertheless, FeNO levels have been reported to be influenced by smoking and other factors [12, 55]. In our previous studies of FeNO detection in female and male volunteers, relatively low mean levels of FeNO were generally detected in the exhaled breath of women and smokers. Considering the large sex differences in smoking prevalence and lesser use of tobacco in women [60], we can confirm the small or absent effect of smoking on FeNO detection in women. The absence of confounder effects when NO is detected in women is an advantage when considering the development of NO‐based diagnostic approaches for early detection of BC.

In the case of menopause, the statistical analysis suggested a borderline significance. Low estrogen promotes metabolic dysfunction in post‐menopause with elevation of pro‐inflammatory cytokines such as IL1, IL6, and TNF‐alpha and consequent increasing of inflammation processes. The mechanisms by which estrogen interferes with the cytokine machinery potentially include the modulation of NO activity [61]. It can be speculated that the estrogen‐driven metabolic and immunological alterations in post‐menopause are linked to NO levels and activities at tissue and systemic levels.

The current POC instrumentation for FeNO detection was developed in clinical practice to detect peaks of exhaled NO during asthma exacerbations. However, FeNO levels in breath measured in this study were in large part around normal physiological values and, additionally, FeNO levels in women's breath are generally lower in comparison to men [12, 55].

To overcome the limitations related to the explorative nature of the current study, more precise detection and sensitive instrumentation that is capable of a wider dynamic range of measurements is required that will enable more detailed analysis of the exhaled NO profile in women. In addition, cohorts of women, including aggressive BC types that seem to be more influenced by NO activity, may provide a comprehensive landscape of FeNO detection in BC. Evaluation of the specificity and relevance of NO in BC needs comparative analysis of NO in respiratory, blood, and cancer tissue together with local and systemic inflammatory markers.

Whether the main limitation of the present study remains the sample size and the imbalance of BC subtypes, the strength and the novelty of this work are based on the intent to meet the clinical diagnostic needs in BC and move to clinical practice by using a well characterized volatile molecule and a POC‐based technology already approved for clinical use, and therefore immediately available. This focuses the efforts of the non‐invasive clinical application of breath analysis on the few well characterized volatile biomarkers with a proven detection method, while awaiting new validated volatile biomarkers from clinical studies of breath analysis currently in progress [22].

Non‐invasive alternatives to breath analysis are addressed to the identification of cells, DNA, and mutations, proteins, or metabolites as potential markers, mainly in blood and urine, but more recently also in saliva, tears, and skin [2, 22]. The advantage of exhaled breath analysis is the total non‐invasiveness of sampling and the nearly unlimited access to samples, but the main disadvantage is the lack of the biological/clinical characterization of volatile compounds in comparison to cells, genes, and proteins [22].

Lastly, the use of the gaseous molecule NO as a diagnostic probe in cancer is at the early stages, and the role of NO in cancer pathophysiology suggests that it has significant potential for translational approaches in addition to the current clinical application in respiratory diseases. Further studies are needed to optimize NO detection in the breath of women with BC and extensively understand the clinical significance and mechanisms underlying the local and systemic alterations of soluble and exhaled NO in cancer and its role as a gaseous and diffusible messenger molecule.

## 5 Conclusion

Our explorative study showed a type‐dependent detection of FeNO in the exhaled breath of women with BC and a low impact of confounders as smoking and age. The lower exhaled NO levels in HER2‐positive BC, although not significant in our study, warrant further investigation, possibly using more sensitive instrumentation and larger cohorts of women. This might help to establish the potential of NO in non‐invasive detection of BC, possibly in conjunction with other volatile BC‐related markers or by extending the NO measurement to blood or tissues.

## Author Contributions

Conceptualization: R.O. and R.M. Data curation: A.P. and M.B. Formal analysis: A.P. Funding acquisition: R.O. Investigation: F.S. and M.B. Methodology: C.V., F.S., and A.P. Supervision: R.O. Writing – original draft: R.O. Writing – review and editing: R.O., F.S., A.P., C.V., R.M., R.A., G.S., and R.B.

## Ethics Statement

The present study was approved by the Ethics Committee of the Fondazione IRCCS Istituto Nazionale dei Tumori (INT 69/18).

## Consent

All breath samples and data were obtained upon written informed consent and according to the institutional rules and the Helsinki declaration.

## Conflicts of Interest

The authors declare no conflicts of interest.

## Supporting information


**Figure S1:** FeNO values and variability of replicate measurements. Each bar is the mean of the 2 replicates obtained by each participant subjected to FeNO breath test. The standard error of the mean is indicated in red.
**Figure S2:** Statistical evaluation of the variability of replicate measurements. Log(FeNO) levels in the first and second replicates obtained by each participant subjected to FeNO breath test are shown. The median of the distribution of the experimental FeNO replicate 1 and 2 were compared and no significant difference was found as indicated by the result of the Wilcoxon test (*p* = 0.25).

**Table S1:** FeNO levels and characteristics of each participant to the study. Personal and anthropometric data, lifestyle factors, and medical history that was focused on chronic disease such as asthma, diabetes and hypertension are reported for the 78 women with early‐stage BC and the 114 control women with negative BC imaging (BI‐RADS = 1, control; BI‐RADS = 2, control with benign disease). Sub‐typing of BC samples according to immunohistochemical determination of hormonal receptors, HER2, and Ki‐67 is also reported.
**Table S2:** Lists the outliers determined by the Grubbs test.

## Data Availability

The data that supports the findings of this study are available in the Supporting Information of this article.

## References

[1] G. B. Hanna , P. R. Boshier , S. R. Markar , and A. Romano , “Accuracy and Methodologic Challenges of Volatile Organic Compound‐Based Exhaled Breath Tests for Cancer Diagnosis: A Systematic Review and Meta‐Analysis,” JAMA Oncology 5, no. 1 (2019): e182815, 10.1001/jamaoncol.2018.2815. 30128487 PMC6439770

[2] G. Trecate , P. M. Sinues , and R. Orlandi , “Noninvasive Strategies for Breast Cancer Early Detection,” Future Oncology 12, no. 11 (2016): 1395–1411, 10.2217/fon-2015-0071. 27044539

[3] P. P. Oza and K. Kashfi , “The Triple Crown: NO, CO, and H2S in Cancer Cell Biology,” Pharmacology & Therapeutics 249 (2023): 108502, 10.1016/j.pharmthera.2023.108502. 37517510 PMC10529678

[4] S. Antonowicz , Z. Bodai , T. Wiggins , et al., “Endogenous Aldehyde Accumulation Generates Genotoxicity and Exhaled Biomarkers in Esophageal Adenocarcinoma,” Nature Communications 12, no. 1 (2021): 1454, 10.1038/s41467-021-21800-5. PMC7935981 33674602

[5] J. O. Lundberg and E. Weitzberg , “Nitric Oxide Signaling in Health and Disease,” Cell 185, no. 16 (2022): 2853–2878, 10.1016/j.cell.2022.06.010. 35931019

[6] R. F. Furchgott and J. V. Zawadzki , “The Obligatory Role of Endothelial Cells in the Relaxation of Arterial Smooth Muscle by Acetylcholine,” Nature 288, no. 5789 (1980): 373–376, 10.1038/288373a0. 6253831

[7] T. Infante , D. Costa , and C. Napoli , “Novel Insights Regarding Nitric Oxide and Cardiovascular Diseases,” Angiology 72, no. 5 (2021): 411–425, 10.1177/0003319720979243. 33478246

[8] D. Fukumura , S. Kashiwagi , and R. K. Jain , “The Role of Nitric Oxide in Tumour Progression,” Nature Reviews. Cancer 6, no. 7 (2006): 521–534, 10.1038/nrc1910. 16794635

[9] P. Panneerselvan , K. Vasanthakumar , K. Muthuswamy , V. Krishnan , and S. Subramaniam , “Insights on the Functional Dualism of Nitric Oxide in the Hallmarks of Cancer,” Biochimica Et Biophysica Acta. Reviews on Cancer 1878, no. 6 (2023): 189001, 10.1016/j.bbcan.2023.189001. 37858621

[10] L. E. Gustafsson , A. M. Leone , M. G. Persson , N. P. Wiklund , and S. Moncada , “Endogenous Nitric Oxide Is Present in the Exhaled Air of Rabbits, Guinea Pigs and Humans,” Biochemical and Biophysical Research Communications 181, no. 2 (1991): 852–857, 10.1016/0006-291x(91)91268-h. 1721811

[11] S. A. Kharitonov , D. Yates , R. A. Robbins , R. Logan‐Sinclair , E. A. Shinebourne , and P. J. Barnes , “Increased Nitric Oxide in Exhaled Air of Asthmatic Patients,” Lancet 343, no. 8890 (1994): 133–135, 10.1016/s0140-6736(94)90931-8. 7904001

[12] R. A. Dweik , P. B. Boggs , S. C. Erzurum , et al., “An Official ATS Clinical Practice Guideline: Interpretation of Exhaled Nitric Oxide Levels (FENO) for Clinical Applications,” American Journal of Respiratory and Critical Care Medicine 184, no. 5 (2011): 602–615, 10.1164/rccm.9120-11ST. 21885636 PMC4408724

[13] H. Zhou , J. Li , Z. Chen , Y. Chen , and S. Ye , “Nitric Oxide in Occurrence, Progress and Therapy of Lung Cancer: A Systemic Review and Meta‐Analysis,” BMC Cancer 21, no. 1 (2021): 678, 10.1186/s12885-021-08430-2. 34103000 PMC8188673

[14] H. Sung , J. Ferlay , R. L. Siegel , et al., “Global Cancer Statistics 2020: GLOBOCAN Estimates of Incidence and Mortality Worldwide for 36 Cancers in 185 Countries,” CA: A Cancer Journal for Clinicians 71, no. 3 (2021): 209–249, 10.3322/caac.21660. 33538338

[15] L. E. Pace and N. L. Keating , “A Systematic Assessment of Benefits and Risks to Guide Breast Cancer Screening Decisions,” Journal of the American Medical Association 311, no. 13 (2014): 1327–1335, 10.1001/jama.2014.1398. 24691608

[16] S. Mook , L. J. Van 't Veer , E. J. Rutgers , et al., “Independent Prognostic Value of Screen Detection in Invasive Breast Cancer,” Journal of the National Cancer Institute 103, no. 7 (2011): 585–597, 10.1093/jnci/djr043. 21350218

[17] H. D. Nelson , R. Fu , A. Cantor , M. Pappas , M. Daeges , and L. Humphrey , “Effectiveness of Breast Cancer Screening: Systematic Review and Meta‐Analysis to Update the 2009 U.S. Preventive Services Task Force Recommendation,” Annals of Internal Medicine 164, no. 4 (2016): 244–255, 10.7326/M15-0969. 26756588

[18] K. S. Peairs , Y. Choi , R. W. Stewart , and H. F. Sateia , “Screening for Breast Cancer,” Seminars in Oncology 44, no. 1 (2017): 60–72, 10.1053/j.seminoncol.2017.02.004. 28395765

[19] K. Lång , V. Josefsson , A. M. Larsson , et al., “Artificial Intelligence‐Supported Screen Reading Versus Standard Double Reading in the Mammography Screening With Artificial Intelligence Trial (MASAI): A Clinical Safety Analysis of a Randomised, Controlled, Non‐Inferiority, Single‐Blinded, Screening Accuracy Study,” Lancet Oncology 24, no. 8 (2023): 936–944, 10.1016/S1470-2045(23)00298-X. 37541274

[20] W. Ibrahim , L. Carr , R. Cordell , et al., “Breathomics for the Clinician: The Use of Volatile Organic Compounds in Respiratory Diseases,” Thorax 76, no. 5 (2021): 514–521, 10.1136/thoraxjnl-2020-215667. 33414240 PMC7611078

[21] D. Y. Graham , P. D. Klein , D. J. Evans, Jr. , et al., “ *Campylobacter pylori* Detected Noninvasively by the 13C‐Urea Breath Test,” Lancet 1, no. 8543 (1987): 1174–1177, 10.1016/s0140-6736(87)92145-3. 2883491

[22] F. Segrado , A. Romano , and R. Orlandi , “Changes in the Volatome of Cancer Patients,” in Volatile Organic Compounds, ed. A. Picciariello and D. F. Altomare (Springer, 2025), 10.1007/978-3-031-83406-6_2.

[23] M. Leemans , P. Bauër , V. Cuzuel , E. Audureau , and I. Fromantin , “Volatile Organic Compounds Analysis as a Potential Novel Screening Tool for Breast Cancer: A Systematic Review,” Biomarker Insights 17 (2022): 11772719221100709, 10.1177/11772719221100709. 35645556 PMC9134002

[24] J. R. Huddy , M. Z. Ni , S. R. Markar , and G. B. Hanna , “Point‐Of‐Care Testing in the Diagnosis of Gastrointestinal Cancers: Current Technology and Future Directions,” World Journal of Gastroenterology 21, no. 14 (2015): 4111–4120, 10.3748/wjg.v21.i14.4111. 25892860 PMC4394071

[25] L. Zhongzhou , Y. Jun , D. Diandian , et al., “E‐Nose Based on a High‐Integrated and Low‐Power Metal Oxide Gas Sensor Array,” Sensors and Actuators B: Chemical 380 (2023): 133289, 10.1016/j.snb.2023.133289.

[26] V. A. Binson , M. Subramoniam , and L. Mathew , “Discrimination of COPD and Lung Cancer From Controls Through Breath Analysis Using a Self‐Developed e‐Nose,” Journal of Breath Research 15, no. 4 (2021): 046003, 10.1088/1752-7163/ac1326. 34243176

[27] V. A. Binson , M. Subramoniam , and L. Mathew , “Prediction of Lung Cancer With a Sensor Array Based e‐Nose System Using Machine Learning Methods,” Microsystem Technologies 30 (2024): 1421–1434, 10.1007/s00542-024-05656-5.

[28] V. A. Binson , M. Subramoniam , and L. Mathew , “Detection of COPD and Lung Cancer With Electronic Nose Using Ensemble Learning Methods,” Clinica Chimica Acta 523 (2021): 231–238, 10.1016/j.cca.2021.10.005. 34627826

[29] J. Jovel and R. Greiner , “An Introduction to Machine Learning Approaches for Biomedical Research,” Frontiers in Medicine 8 (2021): 771607, 10.3389/fmed.2021.771607. 34977072 PMC8716730

[30] C. Park , C. C. Took , and J. K. Seong , “Machine Learning in Biomedical Engineering,” Biomedical Engineering Letters 8, no. 1 (2018): 1–3, 10.1007/s13534-018-0058-3. 30603186 PMC6208556

[31] V. A. Binson , S. Thomas , M. Subramoniam , J. Arun , S. Naveen , and S. Madhu , “A Review of Machine Learning Algorithms for Biomedical Applications,” Annals of Biomedical Engineering 52, no. 5 (2024): 1159–1183, 10.1007/s10439-024-03459-3. 38383870

[32] M. Wieczorek , A. Weston , M. Ledenko , J. N. Thomas , R. Carter , and T. Patel , “A Deep Learning Approach for Detecting Liver Cirrhosis From Volatolomic Analysis of Exhaled Breath,” Frontiers in Medicine 9 (2022): 992703, 10.3389/fmed.2022.992703. 36250077 PMC9556819

[33] H. Hong , J. Sun , and W. Cai , “Multimodality Imaging of Nitric Oxide and Nitric Oxide Synthases,” Free Radical Biology & Medicine 47, no. 6 (2009): 684–698, 10.1016/j.freeradbiomed.2009.06.011. 19524664

[34] X. Li , H. Chen , Y. Wang , H. Chen , and Y. Gao , “BODIPY‐Based NO Probe for Macrophage‐Targeted Immunotherapy Response Monitoring,” Analytical Chemistry 95, no. 18 (2023): 7320–7328, 10.1021/acs.analchem.3c00409. 37113062

[35] M. Maniscalco , C. Vitale , A. Vatrella , A. Molino , A. Bianco , and G. Mazzarella , “Fractional Exhaled Nitric Oxide‐Measuring Devices: Technology Update,” Medical Devices (Auckland, N.Z.) 9 (2016): 151–160, 10.2147/MDER.S91201. 27382340 PMC4922771

[36] X. Wang , X. Kang , X. Chen , et al., “Pocket Electronic Nose Integrating an Ultra‐Compact Sensor Array Chip and Spatiotemporal Network Enables Highly Selective Gas Sensing,” ACS Sensors (2025), 10.1021/acssensors.5c01829. 40857708

[37] L. Liberman and J. H. Menell , “Breast Imaging Reporting and Data System (BI‐RADS),” Radiologic Clinics of North America 40, no. 3 (2002): 409–430, 10.1016/s0033-8389(01)00017-3. 12117184

[38] M. Högman , “Reference Equations for Exhaled Nitric Oxide‐What Is Needed?,” Journal of Breath Research 18, no. 3 (2024): 031001, 10.1088/1752-7163/ad4aba. 38740047

[39] I. Horváth , P. J. Barnes , S. Loukides , et al., “A European Respiratory Society Technical Standard: Exhaled Biomarkers in Lung Disease,” European Respiratory Journal 49, no. 4 (2017): 1600965, 10.1183/13993003.00965-2016. 28446552

[40] V. A. Binson , M. Subramoniam , and L. Mathew , “Noninvasive Detection of COPD and Lung Cancer Through Breath Analysis Using MOS Sensor Array Based e‐Nose,” Expert Review of Molecular Diagnostics 21, no. 11 (2021): 1223–1233, 10.1080/14737159.2021.1971079. 34415806

[41] American Thoracic Society; European Respiratory Society , “ATS/ERS Recommendations for Standardized Procedures for the Online and Offline Measurement of Exhaled Lower Respiratory Nitric Oxide and Nasal Nitric Oxide, 2005,” American Journal of Respiratory and Critical Care Medicine 171, no. 8 (2005): 912–930, 10.1164/rccm.200406-710ST. 15817806

[42] J. D. Pleil , J. D. Beauchamp , R. A. Dweik , and T. H. Risby , “Breath Research in Times of a Global Pandemic and Beyond: The Game Changer,” Journal of Breath Research 14, no. 4 (2020): 040202, 10.1088/1752-7163/abb99a. 33021207

[43] D. J. Best and D. E. Roberts , “Algorithm AS 89: The Upper Tail Probabilities of Spearman's Rho,” Applied Statistics 24, no. 3 (1975): 377, 10.2307/2347111.

[44] T. J. Hastie , Statistical Models in S, 1st ed. (Routledge, 1992), 10.1201/9780203738535.

[45] D. F. Bauer , “Constructing Confidence Sets Using Rank Statistics,” Journal of the American Statistical Association 67, no. 339 (1972): 687–690, 10.1080/01621459.1972.10481279.

[46] M. B. Brown and A. B. Forsythe , “Robust Tests for the Equality of Variances,” Journal of the American Statistical Association 69, no. 346 (1974): 364–367, 10.2307/2285659.

[47] J. P. Royston , “An Extension of Shapiro and Wilk's W Test for Normality to Large Samples,” Journal of the Royal Statistical Society. Series C, Applied Statistics 31, no. 2 (1982): 115–124, 10.2307/2347973.

[48] M. J. Maher , Plots, Transformations and Regression: An Introduction to Graphical Methods of Diagnostic Regression Analysis (Oxford University Press, 1987).

[49] F. E. Grubbs , “Sample Criteria for Testing Outlying Observations,” Annals of Mathematical Statistics 21, no. 1 (1950): 27–58, http://www.jstor.org/stable/2236553.

[50] A. Agresti and M. Kateri , “Categorical Data Analysis,” in International Encyclopedia of Statistical Science, ed. M. Lovric (Springer; Heidelberg, 2011), 10.1007/978-3-642-04898-2_161.

[51] A. Trama , R. Boffi , P. Contiero , et al., “Trends in Lung Cancer and Smoking Behavior in Italy: An Alarm Bell for Women,” Tumori 103, no. 6 (2017): 543–550, 10.5301/tj.5000684. 28967091 PMC6379803

[52] A. Virdis , R. M. Bruno , M. F. Neves , G. Bernini , S. Taddei , and L. Ghiadoni , “Hypertension in the Elderly: An Evidence‐Based Review,” Current Pharmaceutical Design 17, no. 28 (2011): 3020–3031, 10.2174/138161211798157711. 21861835

[53] L. Blanchet , A. Smolinska , A. Baranska , et al., “Factors That Influence the Volatile Organic Compound Content in Human Breath,” Journal of Breath Research 11, no. 1 (2017): 016013, 10.1088/1752-7163/aa5cc5. 28140379

[54] A. Uppalapati , S. Gogineni , and J. R. Espiritu , “Association Between Body Mass Index (BMI) and Fraction of Exhaled Nitric Oxide (FeNO) Levels in the National Health and Nutrition Examination Survey (NHANES) 2007–2010,” Obesity Research & Clinical Practice 10, no. 6 (2016): 652–658, 10.1016/j.orcp.2015.11.006. 26774499

[55] M. Högman , A. Thornadtsson , K. Bröms , et al., “Different Relationships Between F_E_NO and COPD Characteristics in Smokers and Ex‐Smokers,” COPD 16, no. 3–4 (2019): 227–233, 10.1080/15412555.2019.1638355. 31357875

[56] W. T. Walker , A. Liew , A. Harris , J. Cole , and J. S. Lucas , “Upper and Lower Airway Nitric Oxide Levels in Primary Ciliary Dyskinesia, Cystic Fibrosis and Asthma,” Respiratory Medicine 107, no. 3 (2013): 380–386, 10.1016/j.rmed.2012.11.021. 23290188

[57] H. Grasemann , N. Knauer , R. Büscher , K. Hübner , J. M. Drazen , and F. Ratjen , “Airway Nitric Oxide Levels in Cystic Fibrosis Patients Are Related to a Polymorphism in the Neuronal Nitric Oxide Synthase Gene,” American Journal of Respiratory and Critical Care Medicine 162, no. 6 (2000): 2172–2176, 10.1164/ajrccm.162.6.2003106. 11112133

[58] R. A. Mosquera , C. L. Samuels , T. S. Harris , et al., “Decreased Exhaled Nitric Oxide Levels in Patients With Mitochondrial Disorders,” Open Respiratory Medicine Journal 26 (2013): 67–70, 10.2174/1874306401307010067. PMC3735920 23935767

[59] G. Ren , X. Zheng , M. Bommarito , et al., “Reduced Basal Nitric Oxide Production Induces Precancerous Mammary Lesions via ERBB2 and TGFβ,” Scientific Reports 9, no. 1 (2019): 6688, 10.1038/s41598-019-43239-x. 31040372 PMC6491486

[60] WHO Global Report on Trends in Prevalence of Tobacco Use 2000–2025, 3rd ed. (World Health Organization, 2024), accessed October 11, 2024, https://www.who.int/publications/i/item/who‐global‐report‐on‐trends‐in‐prevalence‐of‐tobacco‐use‐2000‐2025‐third‐edition.

[61] J. Pfeilschifter , R. Köditz , M. Pfohl , and H. Schatz , “Changes in Proinflammatory Cytokine Activity After Menopause,” Endocrine Reviews 23, no. 1 (2002): 90–119, 10.1210/edrv.23.1.0456. 11844745

