# Supplementary material for: Explorative Detection of Fractional Exhaled Nitric Oxide (FeNO) in Exhaled Breath of Patients With Breast Cancer
Source: Cancer Med. 2025 Sep 29;14(19):e71279. doi: 10.1002/cam4.71279 (PMC12477705; doi:10.1002/cam4.71279)

**Supplementary Figure 1. Values and variability of replicate measurements.** Each bar is the mean of the 2 replicates obtained by each participant subjected to FeNO breath test. The standard error of the mean is indicated in red.

**Supplementary Figure 2. Statistical evaluation of the variability of replicate measurements.** Log(FeNO) levels in the first and second replicates obtained by each participant subjected to FeNO breath test are shown. The median of the distribution of the experimental FeNO replicate 1 and 2 were compared and no significant difference was found as indicated by the result of the Wilcoxon test (p=0.25).


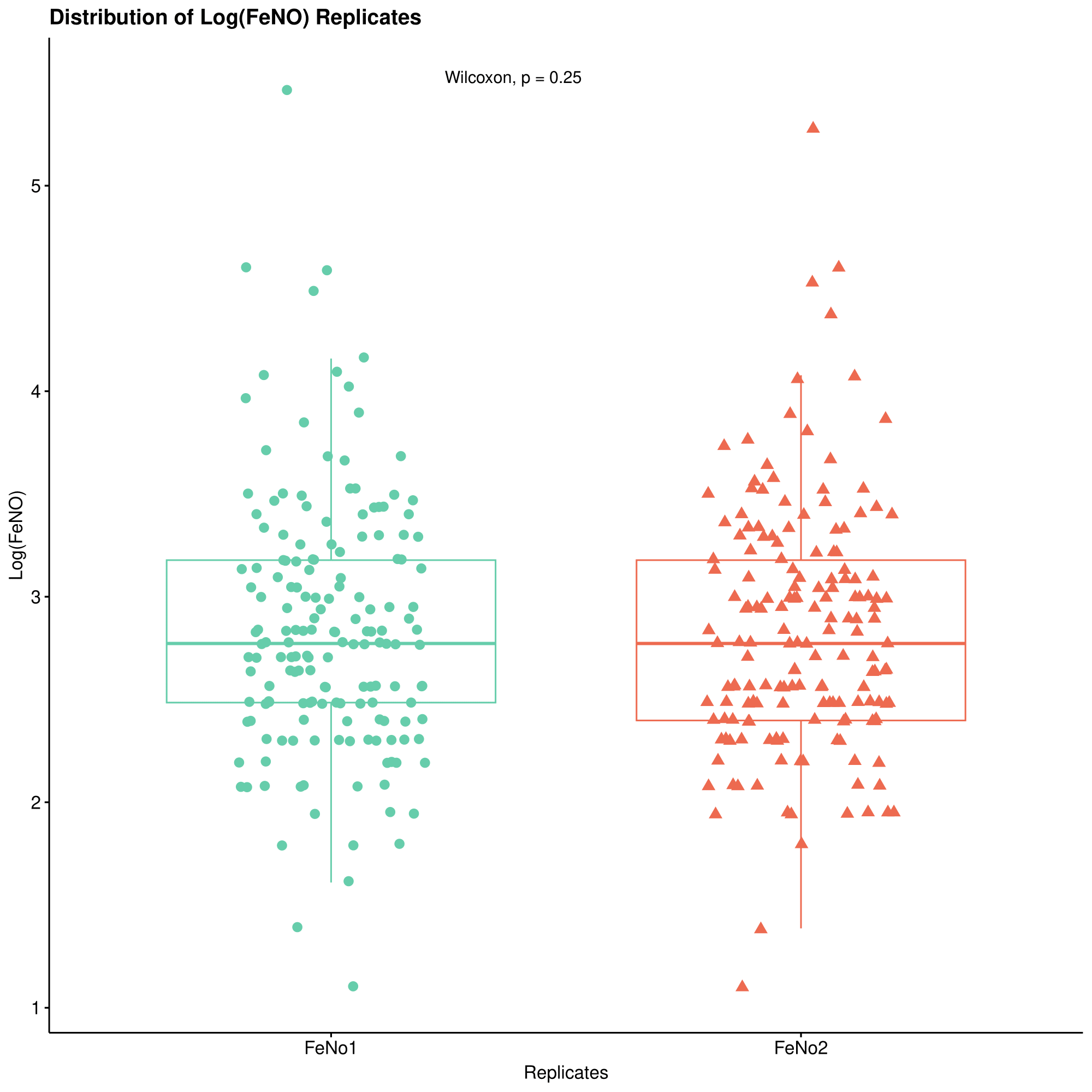

Supplement: Supplementary file 1 — Figure S1: FeNO values and variability of replicate measurements. Each bar is the mean of the 2 replicates obtained by each participant subjected to FeNO breath test. The standard error of the mean is indicated in red. Figure S2: Statistical evaluation of the variability of replicate measurements. Log(FeNO) levels in the first and second replicates obtained by each participant subjected to FeNO breath test are shown. The median of the distribution of the experimental FeNO replicate 1 and 2 were compared and no significant difference was found as indicated by the result of the Wilcoxon test (p = 0.25). [file CAM4-14-e71279-s001.docx]
